# Queen Bees: How Is Female Managers’ Happiness Determined?

**DOI:** 10.3389/fpsyg.2022.741576

**Published:** 2022-02-16

**Authors:** Ailun Xiong, Senmao Xia, Qing Wang, Joan Lockyer, Dongmei Cao, Hans Westlund, Hongyi Li

**Affiliations:** ^1^Research Center for Enterprise Management, Chongqing Technology and Business University, Chongqing, China; ^2^International Centre for Transformational Entrepreneurship and Center for Business in Society, Coventry University, Coventry, United Kingdom; ^3^Warwick Business School, The University of Warwick, Coventry, United Kingdom; ^4^School of Strategy and Leadership, Coventry Business School, Coventry, United Kingdom; ^5^Department of Urban Planning and Environment, School of Architecture and the Built Environment, Royal Institute of Technology, Stockholm, Sweden; ^6^Department of Decision Sciences and Managerial Economics, The Chinese University of Hong Kong, Sha Tin, Hong Kong SAR, China

**Keywords:** subjective happiness, queen bee, female managers, gender-egalitarian, leadership

## Abstract

This paper aims to study the determinants of subjective happiness among working females with a focus on female managers. Drawn on a large social survey data set (*N* = 10470) in China, this paper constructs gender development index at sub-national levels to study how institutional settings are related to female managers’ happiness. We find that female managers report higher levels of happiness than non-managerial employees. However, the promoting effect is contingent on individual characteristics and social-economic settings. The full sample regression suggests that female managers behaving in a masculine way generally report a high level of happiness. Meanwhile, female managers who refuse to support gender equality report low happiness levels. Sub-sample analysis reveals that these causalities are conditioned on regional culture. Masculine behavior and gender role orientation significantly predict subjective happiness only in gender-egalitarian regions. This study is one of the first to consider both internal (individual traits) and external (social-economic environment) factors when investigating how female managers’ happiness is impacted. Also, this study challenges the traditional wisdom on the relationship between female managers’ job satisfaction and work-home conflict. This study extends the literature by investigating the impacts of female managers’ masculine behavior on their happiness. This study is useful for promoting female managers’ leadership effectiveness and happiness.

## Introduction

Research on gender differences in subjective happiness has received substantial attention in early (Røysamb et al., 2002; Bailyn, 2003; Reid, 2004) and more recent studies (Meisenberg and Woodley, 2015; Tao et al., 2018). According to the traditional gender division of labor, women bear the primary responsibility for household work, and men should focus on career development. Therefore females seeking career achievement may suffer more from work-home conflict and distress and overwhelm (Trzcinski and Holst, 2012; Meer, 2014; Chui and Wong, 2016). However, not all women are impacted by work-home conflicts, as some factors, such as cultural context, gender role orientation and role segmentation etc., might mediate this effect (Putnik et al., 2018). Some females may view the work role more importantly or do not segment work from their family. They will not blame the work domains when it conflicts with home domains. That is why some previous studies also suggested that female managers are not necessarily associated with decreased job/life satisfaction (Zhao et al., 2017, 2019).

In leadership science, female managers who attach little importance to the family domain or display dominance in the work domain can be classified as “Queen Bee” (QB). They distance themselves from general women behave like their male counterparts (Derks et al., 2011, 2016; Arvate et al., 2018; Faniko et al., 2020). Social identity theory suggests that people tend to be drawn to those who share the same demographic characteristics. The so-called implicit bias makes people feel comfortable when interacting with similar people (Peterson and Stewart, 2020). Therefore, in a highly masculine environment, QBs are attracted to higher authorities and are more likely to be promoted. After all, selecting a masculine woman into a management position will not challenge the current gender hierarchy (Faniko et al., 2017). On the other hand, a QB is less likely to gain support from her subordinates. Displaying masculine traits may extend physiological distance with female employees (Ely, 1994; Derks et al., 2016).

Above all, previous scholars have not reached a consensus on the determinants of subjective happiness for females in the workplace, especially those who hold managerial positions. This paper seeks to address this question with a large data set in China. Specifically, it aims to answer the following research questions: Does occupational hierarchy matter for females’ subjective happiness? Will female managers report higher levels of happiness when displaying masculine traits? Will context factors such as gender equality play a role in determining female managers’ subjective happiness?^1^ This research tends to contribute to the extant literature in the following ways. First, it is one of the first to construct a complete framework to investigate the impacts of internal factors (personal traits) and external factors (social-economic environment) on QB syndrome. In reality, the external factors are of the same significance as internal factors to explain female managers’ happiness. Second, this study challenges traditional wisdom on the relationship between occupational hierarchy and life/job satisfaction by presenting a new idea in the Chinese context. According to Gender Inequality Index released by the World Bank^2^, China ranked 106th among 187 economies. China has achieved gender equality in some domain, but still lags behind in some other domains (Liu et al., 2014). The Chinese unique social and economic settings thus may affect females’ attitudes on leadership preferences. Previous studies mainly focus on the western world and the significance of cultural context have been relatively overlooked. The previous assumption derived from QBs theory is based on either egalitarian regions or inegalitarian regions. The significance of cultural context has been relatively overlooked and it will be valuable to further test QB theory with observations in China. Finally, this study extends the traditional literature by investigating the impacts of female managers’ masculine behavior on their happiness. Previous studies usually limit themselves to the influence of masculinity behavior on work effectiveness.

The rest of the paper is organized as follows. Section two briefly reviews the literature on QBs and gender inequality. Research hypotheses are also elaborated in this section. Section three presents the empirical strategy and the data set, model and variables. Section four presents the estimation results and highlights the main empirical findings. Concluding remarks are summarized in the final section.

## Literature Review and Research Hypotheses

### Occupational Hierarchy and Subjective Happiness

The pursuit of happiness is a major motivation behind human behaviors. Previous scholars identify at least four theories that help explain the individual difference in subjective happiness (Larsson and Thulin, 2018). The *goal theory* implies that the fulfillment of personal goals contributes to subjective well-being. People who fail to fulfill their goals or to reach a goal that is not in line with their needs report lower levels of subjective happiness (Diener et al., 1999). *Activity-based theory* suggests that happiness is a function of actions and behaviors. People will not experience higher happiness levels if their tasks are either too easy or too difficult (Diener et al., 1999). *Personality theory* indicates that certain personalities (e.g., Extraversion or optimism) should be a powerful predictor of subjective happiness (DeNeve and Cooper, 1998). Finally, the discrepancy theories hold that happiness stems from the comparison with other people or previous states (Diener et al., 1999). Therefore, at least 30 social-demographic, psychological or institutional factors may contribute to subjective happiness (Dolan et al., 2008).

Based on the framework mentioned above, we believe that people at the top of the occupational hierarchy will report higher levels of subjective happiness (Trzcinski and Holst, 2012). On the one hand, a higher occupational hierarchy is positively associated with personal income, which can be utilized to fulfill various needs (e.g., food, good accommodation, and luxuries). This is why wealth is the most potent determinant of subjective happiness (Mentzakis and Moro, 2009). On the other hand, Individuals in management positions receive more respect and admiration, have a strong feeling of power and acceptance, and are able to control and influence others’ decisions (Anderson et al., 2012). This may create a sense of superiority compared with general employees^3^.

However, previous studies also have noticed certain gender differences regarding the promoting effect of the occupational hierarchy (Eagly and Karau, 2002; Rudman and Fairchild, 2004). Not all individuals attach importance to career success. Traditional women care more about the family domain. Holding management positions may also occupy their time to take care of the family. This is why job security sometimes exerts a limited impact on female employees’ subjective happiness (Huang et al., 2019). It has also been found that housewives are happier than full time working females (Okulicz-Kozaryn and da Rocha Valente, 2018). Therefore, female managers may not necessarily report higher levels of subjective happiness than employees like males do when they have been promoted.

With regard to China, we believe that the occupational ladder effect outweighs the gender disparities effect. According to Aaltion and Huang (2007), Chinese females view their career success as a way to achieve high social status and economic independence. The desire for self-fulfillment is as equally vital as their male counterpart do. More importantly, family roles and work roles are not two distinctive domains in China (Liu et al., 2013). In a collectivistic culture, the commitment to work is viewed as a means to enhance the family financial situation. Female career advancement is also beneficial to the whole family (Yang et al., 2000). Last but not least, grandparents traditionally help with childcare in China, which relieve the pressure females face on work-family conflict (Liu et al., 2013). Therefore, more and more women have moved up the career ladder and got a high income (Ong et al., 2020). Based on a nationwide survey, Luo (2016) indicated that achieved status and job characteristics have a similar association with job satisfaction for both genders in China. In a more recent study, Xiang et al. (2016) found that power and prestige derived from occupational stratification are the strongest predictors of subjective happiness, and this promoting effect will not be mediated by demographic factors such as gender and age. Taking all these into considerations, we propose that:

**Hypothesis 1**: *Female managers report higher levels of happiness than general employees.*

### Social Identity Threat, Queen Bees and Subjective Happiness

Although female managers generally report higher levels of happiness, the promoting effect of occupational hierarchy will be moderated by a number of individual and context factors. Economic progress in China is not always accompanied by the improved status of female in the labor market (Tatli et al., 2016). Women’s labor force participation rate dropped 10 percent in the last 20 years (Liu et al., 2013). Only 10% of board members are female (CS Gender, 2014). Compared with men, females are less able to be perceived as legitimate authorities (Eagly and Karau, 2002; Rudman and Fairchild, 2004). Personality traits of qualified leaders, such as confidence, decisiveness and assertiveness, are more closely related to males than females (Lyness and Heilman, 2006). Being a powerful woman in China still violate gender stereotypes, which results in various forms of threats and penalties (Morris and Daniel, 2008; Heilman and Wallen, 2010; Williams and Tiedens, 2016). Therefore, women who are similar to men in leadership style and personalities are more likely to be promoted. To achieve better career development, some female leaders may display excessive masculine traits and distance themselves from other women, a phenomenon which is known as “Queen Bee” (Derks et al., 2016).

According to Derks et al. (2016), a QB is characterized by several traits. The first is to legitimize gender inequality (Powell et al., 2009). For instance, Stroebe et al. (2009) revealed that successful women in male-dominated settings agree with gender-biased selection procedures. Based on a questionnaire in Hong Kong, Ng and Chiu (2001) found that junior women support favorable policies for female career development. Senior women, however, hold less favorable views toward these policies. A recent Chinese survey points out that women in higher positions refuse to admit the existence of gender discrimination (Zhilian Recruiting, 2017). Conversely, women in lower positions believe that gender discrimination is a common phenomenon in the workplace (Zhilian Recruiting, 2017). Second, QBs also tend to present themselves in a masculine way, that is, to emphasize characteristics associated with males. Several studies indicated that senior female faculty describe themselves as equally masculine as their male counterparts in terms of being assertive, competitive and risk-taking (Derks et al., 2016). Female board members in European companies are also more status-oriented - a typical masculine characteristic than men (Derks et al., 2011).

The social identity theory explains why and how QBs emerged in the workplace. People are always a part of certain social groups. Group memberships determine who we are and what we should do. Positive self-esteem and confidence can be derived from high-status group membership. However, those in low group status will take necessary initiatives to avoid adverse psychological outcomes, such as joining the other group (Scheepers and Ellemers, 2019). For instance, a fan of a relegated football team may turn to support other teams to obtain positive feelings. However, the boundary of gender is less permeable. Female managers can only realize group mobility by acting like their male counterparts (Scheepers and Ellemers, 2019). This is why some female leaders are even more aggressive and decisive than males (Derks et al., 2016).

Queen bee behaviors produce a divergent impact on subjective happiness. On the one hand, female leaders who display typical QB traits are more likely to be selected into powerful positions, especially in male-dominated settings. Due to the call for gender equality, many countries and organizations face pressure to promote female leaders (Krook, 2016; Sojo et al., 2016). Selecting a QB into management positions alleviates the external stress of meeting the quota for women in leadership without jeopardizing the current gender hierarchy (Vial et al., 2016). After all, a QB might deny the existence of gender discrimination and refuse to give opportunities for other women. Therefore, due to better job prospects, QBs should report higher levels of happiness. On the other hand, a QB is less likely to gain support from female subordinates. This should also be negatively related to the subjective happiness of QBs. As aforementioned, QBs distance themselves from junior women and usually hold negative attitudes. Based on samples of over 1,000 corporations in Australia, Gould et al. (2018) found that under certain circumstances, women who have achieved a high-status role refuse to support other females seeking senior positions. Such stereotypical opinions expressed by a female leader are detrimental to junior women (Sutton et al., 2006). Moreover, low-status members support high-status members only when the latter is actively involved with the former (Van Laar et al., 2014). Therefore, it is difficult for a QB to cultivate high-quality leader-subordinate relations.

It should be noted that different dimensions of masculine traits may not emerge simultaneously. Female managers who present themselves as masculine do not necessarily defend the current gender hierarchy. Kelan (2019) indicates that men who support gender equality are associated with better career prospects. Therefore not all male leaders will practice gender bias. Compared with masculine self-presenting, gender inequality legitimization exerts a more detrimental effect on female subordinates. Taking all this into consideration, we propose that

**Hypothesis 2**: *Female managers who present themselves in a masculine way are associated with higher happiness levels.*

**Hypothesis 3**: *Female managers who defend gender inequality are associated with lower happiness levels.*

Since legitimization is the major barrier for females in the workplace, the subjective happiness level for female managers will also be moderated by the gender role attitude of the people surrounding them. As summarized by Başlevent and Kirmanoğlu (2017), individuals’ expectation toward work and family is shaped by regional culture. In less egalitarian cultures, women who spend less time in the family domain and more time in the workplace might feel socially excluded as their lives are not in line with social norms. For instance, in a laboratory experiment, McGlashan et al. (1995) found that group members who held traditional stereotypes toward females tended to rate female leaders negatively. With the gradual liberalization of the Chinese economy and the integration of global development, women in China enjoy a greater degree of autonomy than ever before. They are able to find more opportunities to increase their income. Females thus gain greater equality in both home and work task divisions (Tatli et al., 2016). However, China is a large country that consists of 56 ethnic groups. There are also significant economic and social gaps between different regions. For instance, east-coast China is more open, developed and liberalized than non-east regions (Fleisher and Chen, 1997). Thus, female managers will have higher happiness levels in more egalitarian regions. On the other hand, QBs may have lower happiness levels in egalitarian regions as feminine leadership styles are more widely accepted. Therefore, we have the following hypotheses.

**Hypothesis 4**: *Female managers have higher happiness levels in egalitarian regions.*

## Data, Model, and Variables

In an attempt to study the determinants of the subjective happiness of female managers, the logit model is employed with details as follows. The data is obtained from the Chinese General Social Survey (CGSS), the first nationwide survey conducted by Renmin University. CGSS aims to systematically monitor the changing relationship between social structure and quality of life in both urban and rural China. Social structure refers to dimensions of social group and organization as well as networks of social relationships. Quality of life is the objective and subjective aspects of the people well-being both at the individual and aggregate levels^4^.

Chinese General Social Survey data has been widely used in political and social science research in China (e.g., Xiong et al., 2017). Since each respondent is not likely to be sampled repeatedly in different rounds, we combined the data of three waves (2011, 2012, and 2013) to increase the sample size. Respondents that were unemployed, self-employed or at school were removed. Observations with missing data are also deleted. The remaining sample size is 4,239 females with 1,082 managers and 6,221 males with 1,590 managers. According to the information on the official website, mainland China is divided into 2,800 primary sampling units. 125 primary sampling units (PSU) were selected (in each wave) and four secondary-level sampling units are matched with each PSU. Two third-level sampling units are selected for each secondary-level sampling unit. Finally, 10 household are selected for each third-level sample unit. The fourth-level sample units generally refer to communities or streets, and there are 110,000 communities in urban China^5^. Given the huge population size in China, one person will not be interviewed twice. The overall sample size of CGSS is determined by “*n* = (*Z*^2^*S*^2^)/*d*^2^.” The confidence level is set to 0.95 and the standard error is set to 0.03. Therefore, there are 8,000 samples in each wave which is about 0.05‰ of total population.

The variable *Happy* refers to subjective happiness, the dependent variable in this study. Respondents are required to choose between happy = 1 and not happy = 0 when answering the question: Taking all these together, would you say that you are happy or not happy. We understand that this may not be a perfect of life satisfaction. As indicated by Ponocny and Weismayer (2015), there are always some kinds of positive tendencies in self-ratings. People rated as happy in the survey do not necessarily mean that their conditions are excellent. However, people rated as “not happy” must be very unsatisfied with their conditions. A binary dependent variable would help us sort out the least happy people. *MAN* refers to the occupational hierarchy. Respondents who hold management positions were coded as 1 (those responsible for supervising other employees in the workplace); otherwise, they were coded as 0. Although this paper focuses on senior women, samples of junior women are also included to compare.

Since the dependent variable is binary, logit and probit models are used. In an empirical analysis, both models are appropriate in dealing with dichotomous dependent variables, i.e., the probabilities of an event compared with its opposite outcome (e.g., pass/fail or win/lose). Logit models use a logit link function $f\left(\mu_{y}\right)=\ln\left(\frac{p}{1-p}\right)$; while probit regressions use an inverse normal link function *f*(μ_*y*_) = Φ^−1^(*P*). Both models produce similar results, but probit models can be generalized to account for non-constant error variances in more advanced econometric settings. Our baseline model is presented as follows:


$$PR{\left(Happy\right)}_{i}=\alpha_{0}+\alpha_{1}MAN_{i}+\alpha_{2}QB_{i}+\alpha_{3}GII_{i}+\alpha_{4}CV_{i}+\varepsilon_{i}$$

*QB* refers to the queen bee behavior, which is measured through two discrepant but compatible dimensions: gender-role attitudes and masculine traits. In accordance with McHugh and Frieze (2010), the following four items are included to measure gender-role attitudes. (1) Men are supposed to focus on career development and leave house chores to their wives (*CRD*). (2) Men are inherently more outstanding than women (*MOW*). (3) Men should have more privileges than women when jobs are scarce (*JOB*). (4) For women, it is more important to marry the right person than to do a right job (*MRP*). Respondents are required to answer each question based on a 5 point-scales ranging from “completely disagree” (=1) to “completely agree” (=5). Apparently higher scores indicate lower levels of gender-egalitarian.

The CGSS includes a wide range of questions on values, norms, and attitudes. Several items that may reflect gender differences were selected including preferences on news (*NEWS*) and sports (*SPTS*), attitudes toward premarital sex (*PRES*) and extramarital affairs (*EXAF*). According to a report issued by the Pew Research Center in 2008, there is a significant gender difference in news preference. Men are more interested in news on sports, science, political issues, and international relations, whereas women are more concerned about culture, art and entertainment news. Further, males read newspapers more frequently, and females prioritize magazines. Regarding attitudes toward sex, a previous study proposes that males are more permissive than females (Oliver and Hyde, 1993). Due to a large number of influencing factors available, we use the principal component analysis (PCA) to combine the variables. Principal component analysis is a statistical method that simplifies the complexity in high-dimensional data while retaining trends and patterns. All the principal components are orthogonal to each other, so there is no redundant information. The principal components form an orthogonal basis for the space of the data. The PCA results are reported in Table 1 below.

**TABLE 1:** Principal component analysis (PCA) of QB behaviors.

PCA	Cumulative	CRD	MOW	MRP	JOB	SPTS	NEWS	PRES	EXAF
PC1	0.4293	0.5613	0.5214	0.5456	0.5107	−0.1526	−0.2571	0.1525	0.0523
PC2	0.6741	−0.1062	−0.1196	−0.1403	−0.0743	0.5705	0.5108	0.4780	0.5121
PC3	0.7421	0.1048	0.1132	0.0482	0.1569	0.1865	0.1302	0.1970	0.2120
PC4	0.7948	0.2178	0.1149	0.1804	−0.4123	−0.3220	0.3400	0.0349	0.0319
PC5	0.8544	−0.1943	−0.0967	0.0129	0.3788	−0.3658	0.2701	−0.1121	−0.2220
PC6	0.9211	−0.2892	−0.1321	0.3231	0.0312	0.0981	−0.1094	0.1859	0.1101
PC7	0.9781	0.0725	0.0894	−0.1508	0.1944	0.1152	0.1480	−0.5921	−0.5414
PC8	1.0000	0.2841	−0.5421	0.0020	0.1522	0.0240	0.0150	−0.0022	−0.0061

*PC1 and PC2 contain over 67% of information on the overall eight variables. Information on the first four variables mainly reside in the PC1, while information on the last four variables mainly resides in PC2. They are selected as the proxy variables for QB behaviors. The overall sample size is 10,460. Principal components are new variables that are constructed as linear combinations or mixtures of the initial variables. These combinations are done in such a way that most of the information within the initial variables is extracted into fewer components.*

Column 2 shows the cumulative eigenvalues, which is the cumulative amount of information summarized in each component. For instance, PC1 contains 42% of the information about the overall eight variables. PC1 and PC2 together contain 67% of overall information, etc. Column 3 to column 10 shows the extent to which each variable contributes to building the corresponding component. For example, PC1 mainly contains information on *CRD*, *MOW*, *MRP*, and *JOB*, while PC2 consists of the last four variables. It should be noted that each component is uncorrelated with the others, suggesting that to some extent, the two dimensions of QB behaviors are independent. Women who hold a more conservative attitude toward gender equality do not necessarily present themselves masculine and vice versa. We then select the first two components as proxy variables for QB.

*GII* refers to the gender inequality index computed based on the method in the Human Development Report. It measures gender-based disadvantage in three dimensions: (1) reproductive health, (2) empowerment, and (3) labor market. It shows the loss in potential human development due to inequality between female and male achievements in these dimensions. *GII* ranges from 0, where women and men fare equally, to 1, where one gender scores as poorly as possible in all measured dimensions.


$$G_{\bar{F},\bar{M}}=\sqrt[{3}]{\bar{Health}\cdot \bar{Empowerment}\cdot \bar{LFPR}}$$

$$\bar{Health}=\left(\sqrt{\frac{10}{MMR}\cdot \frac{1}{ABR}+1}\right)/2$$

where


$$\bar{Empowerment}=\frac{\left(\sqrt{PR_{F}\cdot SE_{F}}+\sqrt{PR_{M}\cdot SE_{M}}\right)}{2}$$

and *$\sqrt{LFPR}=\frac{LFPR_{F}+LFPR_{M}}{2}$*

According to the Human Development Report, we use *MMR*, *ABR*, *PR*, *SE*, and *LFPR* to refer to maternal mortality ratio, adolescent birth rate, the share of parliamentary seats held by each sex, population with at least secondary education and labor force participation rate. Some of the data are not available in China at regional levels. As a result, we replace them with similar variables. For instance, “the share of parliamentary seats held by each sex” is replaced by “the share of people’s congress seats held by each sex,” and the data can be found at the website of the National People’s Congress of the People’s Republic of China. “Population with at least secondary education” is replaced by “Population with at least high school education,” and the data is retrieved from the Chinese Population and Employment Statistic Yearbook (CPESY). According to this data, up to 2015, around 28% of Chinese citizens have received a high school education. Other data can be obtained from the China Statistic Yearbook.

Table 2 reports *GII* at sub-national levels in China, which reveals the unequal development of gender equality. East coast regions such as Beijing, Jiangsu, and Zhejiang have lower levels of *GII*, suggesting higher levels of gender equality. In less developed regions in western and middle China, such as Gansu, Yunnan, Inner Mongolia, there are relatively higher levels of *GII*. The economic reforms in 1978 produced two divergent results for gender development. With the transformation from state-socialism to market-socialism, some policy arrangements that improved gender equality during Mao’s era have been dismantled. However, the reform also encourages women’s self-awareness, ultimately facilitating women’s development (Min, 2011). Western scholars also claim that economic growth may bring about new forms of gender inequality (Elson, 2009). Economic achievements and gender equality are not always synchronized (Mitra et al., 2015). Therefore, gender development is only partially, not entirely associated with economic growth in China.

**TABLE 2:** GII score of different regions.

Region	Score	Region	Score
Anhui	0.3120	Liaoning	0.2567
Beijing	0.1806	Neimenggu	0.3201
Fujian	0.2606	Ningxia	0.3564
Gansu	0.3392	Qinghai	0.3177
Guangdong	0.2364	Shandong	0.2531
Guangxi	0.2822	Shaanxi	0.2764
Guizhou	0.2979	Shanxi	0.2958
Hebei	0.2520	Shanghai	0.1641
Henan	0.2685	Sichuan	0.2989
Heilongjiang	0.2948	Tianjing	0.1968
Hubei	0.2804	Xingjiang	0.3252
Hunan	0.3025	Yunan	0.3337
Jiling	0.3012	Zhejiang	0.2300
Jiangsu	0.1964	Chongqing	0.3268
Jiangxi	0.3126		

In the model, *CV* refers to a set of control variables closely related to subjective happiness and gender role attitudes (see Table 3 for details). *INC* refers to the self-rated income level on a 5-point scale. Income is significant in predicting happiness due to the improvement of material well-being. Both the absolute income level and relative income level are positively related to happiness (Caporale et al., 2009). Respondents who have been married were coded as “1,” and other marital status (e.g., single, divorced, and widow) were recorded as “0” (*MARR*). It has been found that young women who are more conservative will enter marriage earlier (Barber and Axinn, 1998). Meanwhile, Kaufman and Taniguchi (2010) found that marriage promotes happiness in both eastern and western countries. Education also exerts a significant impact on gender role attitudes. Jayachandran (2015) found that improving overall education level helps remove favoritism toward males. Women who are well educated thus hold a more egalitarian view on gender roles. We thereby coded college degree as “1” and education below college as “0” (*EDU*). Other demographic variables such as self-rated social status (*STA*), age (*AGE*), religious beliefs (*RLB*) are also included.

**TABLE 3:** Explanations on variables.

Code	Variable	Explanation	Descriptive statistics
HAPP	Self-rated happiness	Happy = 0, Not happy = 1	Mean = 0.72, *S.D*. = 0.33
INC	Self-rated relative income level	Far below average level = 1; below average level = 2; average level = 3; above average level = 4; far above average level = 5	Mean = 3.29, *S.D.* = 0.85
MARR	Marital status	Married = 1, Other = 0	Mean = 0.68, *S.D.* = 0.32
EDU	College education	Bachelor degree and above = 1, Other = 0	Mean = 0.38, *S.D.* = 0.29
RLB	Religious belief	Profess belief, Yes = 0, NO = 1	Mean = 0.91, *S.D.* = 0.18
AGE	Age of respondents	Age of respondents	Min = 18, Max = 65 Mean = 41, *S.D.* = 10.44
REL	Frequency of contacting and meeting with relative	Never = 1; seldom = 2; occasionally = 3; often = 4; everyday = 5	Mean = 3.55, *S.D*. = 1.04
STA	Self-rated social status	Lowest level in the country = 1; highest level in the country = 10	Mean = 6.44, *S.D.* = 1.56
MAN	Management position	Responsible for supervising: employees = 1; other = 0	Mean = 0.26, *S.D*. = 0.48
EQU	Gender equality Index at regional level	Gender inequality index based on methods in Human Development Report	Min = 0.16, Max = 0.35 Mean = 0.27, *S.D*. = 0.05
CRD	Men are supposed to focus on career development and leave house chores to their wives	Completely disagree = 1; Disagree = 2; neither agree nor disagree = 3; agree = 4; completely agree = 5	Mean = 3.22, *S.D*. = 0.87
MOW	Men are inherently more outstanding than women	Completely disagree = 1; disagree = 2; neither agree nor disagree = 3; agree = 4; completely agree = 5	Mean = 2.66, *S.D*. = 1.11
MRP	For women, it is more important to marry a right person than to do a right job	Completely disagree = 1; disagree = 2; neither agree nor disagree = 3; agree = 4; completely agree = 5	Mean = 3.02, *S.D*. = 0.94
JOB	Men should have more right to job than women when jobs are scarce	Completely disagree = 1; disagree = 2; neither agree nor disagree = 3; agree = 4; completely agree = 5	Mean = 1.92, *S.D*. = 1.11
SPT	Frequency of watching sports match	Never = 1; seldom = 2; occasional = 3; often = 4; everyday = 5	Mean = 1.63, *S.D*. = 0.88
NEW	Frequency of reading news paper	Never = 1; seldom = 2; occasional = 3; often = 4; everyday = 5	Mean = 2.65, *S.D*. = 1.09
PRES	Attitude toward premarital sex	It is incorrect to do so = 3; neither correct or incorrect = 2; it is correct to do so = 1	Mean = 2.43, *S.D*. = 0.57
EXAF	Attitude toward extramarital affairs	It is incorrect to do so = 3; neither correct or incorrect = 2; it is correct to do so = 1	Mean = 2.79, *S.D.* = 0.66
QB1	Constructed index of gender role attitude	PCA based on JOB, CRD, MOW, MRP	Min = −2.25, Max = 1.89 Mean = −0.26, *S.D.* = 0.78
QB2	Constructed index of masculinity	PCA based on SPT, NEW, PRES, EXAF	Min = −1.98, Max = 2.11 Mean = 0.13, *S.D*. = 0.94
URN	Urban residence	Live in rural area = 1; live in urban area = 0	Mean = 0.27, *S.D*. = 0.36
YEAR	The year of survey	Samples spans from 2011 to 2013	

*The overall sample size is 10,460.*

## Empirical Results and Discussion

In accordance with the hypotheses, we will first conduct an empirical analysis to examine the determinants of subjective happiness for both female and male samples. This would help us understand whether the occupational hierarchy plays a general significant role in promoting happiness.

### Gender Difference on Subjective Happiness

The estimates for the female and male samples are presented in Tables 4, 5, respectively. Three regression techniques are used, including logit models, probit models, and logit models with clustering effects. Most of the variables are significant and exhibit the expected signs. A positive relation between management position and happiness is observed with a coefficient of 0.209 and 0.337 for females and males, respectively. For female managers, the probability of being happy (versus not happy) is 23% [(e^0.209^−1)%] higher than female employees. For male managers, the probability of being happy (versus not happy) is 40% [(e^0.337^−1)%] higher than male employees. Both male and female managers are happier than ordinary employees, which supports our hypothesis, although the probability of feeling happy is more evident for male managers. Social status (*STA*) and relative income level (*INC*) is positively related to subjective happiness for both male and female samples. An increase in one level of the social hierarchy leads to a 17 and 24% increase in the probability of being happy (versus not happy) for females and males, respectively. Similarly, an increase in one level of income leads to 39 and 58% increase in the probability of being happy (versus not happy) for females and males, respectively. This result is consistent with the findings of Mcbride (2001), which offers evidence that one’s relative income does matter for the perception of subjective happiness.

**TABLE 4:** Determinants of subjective happiness (female sample).

	Subjective happiness (female)			
	Model 1	Model 2	Model 3	Model 4
MARR	0.161	0.093	0.161	0.161*
EDU	–0.071	–0.038	–0.071	–0.071
RLB	−0.329**	−0.191**	−0.329**	−0.329***
AGE	–0.004	–0.002	–0.004	–0.004
REL	0.116*	0.063	0.116	0.116**
STA	0.159***	0.086***	0.159***	0.159***
INC	0.331***	0.181***	0.331***	0.331***
MAN	0.209*	0.120*	0.209*	0.209***
LR χ^2^	119.93	119.25	112.34	174.41
N. obs.	4239	4239	4239	4239

***p < 0.001, **p < 0.01, *p < 0.05. Column 1 refers to the regular logit model; column 2 refers to the probit model; column 3 refers to the logit model with clustering effect on surveying time (year); column 4 refers to the logit model with clustering effect on residential areas (URN).

**TABLE 5:** Determinants of subjective happiness (male samples).

	Subjective happiness (male)			
	Model 1	Model 2	Model 3	Model 4
MARR	0.071	0.027	0.071**	0.071***
EDU	0.113	0.031	0.113*	0.113***
RLB	−0.251**	−0.145***	−0.251***	−0.251***
AGE	0.115	–0.032	0.115	0.115**
REL	0.086	0.038	0.086	0.086
STA	0.212***	0.118***	0.212***	0.312***
INC	0.461***	0.203***	0.461***	0.461***
MAN	0.337***	0.121**	0.337***	0.337***
LR χ^2^	181.51	119.25	217.14	229.77
N. obs.	6221	6221	6221	6221

***p < 0.001, **p < 0.01, *p < 0.05. Column 1 refers to the regular logit model; column 2 refers to the probit model; column 3 refers to the logit model with clustering effect on surveying time (year); column 4 refers to the logit model with clustering effect on residential areas (URN).

### Queen Bee Effects on Subjective Happiness

In this section we construct two indicators for QB traits based on the principal component analysis. QB_1_ measures the extent to which a female leader seeks to legitimize gender inequality. QB_2_ measures the extent to which a female leader presents herself in a masculine way. Models 1 through 3 cover samples of women in management positions, and models 4 through 6 involve samples of all female employees.

The results of Table 6 suggest that queen bee behaviors indeed influence subjective happiness. Legitimization of gender inequality reduces the probability of being happy (versus not happy), and displaying masculine traits increase the probability of being happy (column 1). The hypothesis that QB is a multi-faceted concept and exerts divergent impact is supported (Vial et al., 2016). This argument is further examined in models 2 and 3 with the probit model and clustering effects. It should be noted that the significant association observed in models 1 through 3 can be caused by other factors such as personalities. For instance, QB_2_ involves variables about attitudes toward sex. Individuals who prefer an indulgent lifestyle are more open in sexual behavior, which is positively associated with happiness. Models 4 through 6 are thus added to exclude such possibility. The results suggest that for employees, whether defending the current gender hierarchy or not is not significant in predicting subjective happiness. The masculine traits matter for subjective well-being for female managers only. Religious belief (RLB) is significant in predicting happiness for employees rather than managers. Meanwhile, marital status (MARR) is significant for manager groups but insignificant for employees. The determinants of subjective happiness for these two groups vary considerably.

**TABLE 6:** Determinants of subjective happiness (managers and employees).

	Female managers			Female employees		
	Model 1	Model 2	Model 3	Model 4	Model 5	Model 6
MARR	0.658**	0.396**	0.658***	–0.007	–0.001	–0.007
EDU	–0.260	–0.154	–0.260	–0.005	–0.004	–0.005
RLB	–0.297	−0189	–0.297	−0.375***	−0.213***	−0.375***
AGE	0.009	0.055	0.009	–0.002	–0.001	–0.002
REL	0.141	0.077	0.141*	0.106	0.050	0.106
STA	0.185***	0.106***	0.185***	0.150***	0.081***	0.150***
INC	0.221	0.124	0.221	0.381***	0.202***	0.381***
QB_1_	−0.125**	−0.068**	−0.125*	–0.041	–0.023	–0.041
QB_2_	0.122*	0.069*	0.122*	–0.011	–0.048	–0.011
LR χ^2^	42.03	42.29	48.72	75.90	74.75	62.68
N. obs.	1082	1082	1082	3157	3157	3157

***p < 0.001, **p < 0.01, *p < 0.05. Model 1 to model 3 is computed with samples of female managers. Models 4 to 6 refers to samples of female employees. Columns 1 and 4 refer to regular logit models; columns 2 and 5 refer to probit models; columns 4 and 6 refer to logit models with clustering effect on residential areas (URN). There are 1,082 female manager observations and 3,157 employee observations.

### Gender, Happiness and Regional Difference

Hypothesis 5 proposes that the subjective happiness of female managers is contingent on context factors, i.e., gender equality. The *GII* index at the provincial level is constructed to distinguish egalitarian regions. Therefore, this section first divided samples into two groups based on the *GII* scores calculated in Table 2. Female managers may face more barriers in less egalitarian regions (with higher *GII* scores) and fewer barriers in the egalitarian region (with lower *GII* scores).

In Table 7, samples in egalitarian regions are estimated in models 1–3, and samples in less egalitarian regions are estimated in models 4–5. The results suggested that female managers in egalitarian regions are significantly happier than female employees. The differences, however, are insignificant in the non-egalitarian regions. Traditional gender orientation is associated with lower levels of happiness in egalitarian regions. Self-presenting in a masculine way is positively associated with happiness. This confirms our hypothesis that female leaders face stronger discrimination when people hold more traditional views on gender roles. Previous studies found that males are also happier due to gender equality, which may produce a synergistic effect on females’ subjective well-being (Bjørnskov et al., 2007). Although gender discrimination lifts the burden of housework from the males’ shoulders, it will also constrain their career choices and force them to work harder. Therefore, gender equality increases the subjective happiness for males if they prefer the freedom of choice of occupation over the pursuit of high wages (Bjørnskov et al., 2007). Since gender-egalitarian regions in China are generally more open and developed (see Table 2), it is no wonder that females especially female managers, are happy in these regions.

**TABLE 7:** Determinants of happiness (egalitarian regions and less egalitarian regions).

	Egalitarian regions			Less egalitarian regions		
	Model 1	Model 2	Model 3	Model 4	Model 5	Model 6
MARR	0.054	0.025	0.054	0.305	0.181	0.305
EDU	–0.044	–0.023	–0.044	–0.117	–0.053	–0.117
RLB	–0.133	–0.083	–0.133	−0.525***	−0.295***	−0.525***
AGE	0.001	0.001	0.001	–0.001	–0.006	–0.001
REL	0.141	0.075	0.141	0.082	0.045	0.082
STA	0.158**	0.156**	0.158**	0.162***	0.085***	0.162***
INC	0.278***	0.157***	0.278***	0.389***	0.209***	0.389***
MAN	0.306**	0.174**	0.306***	0.081	0.046	0.081
QB_1_	−0.202**	−0.102**	−0.202**	–0.071	–0.027	–0.071
QB_2_	0.172*	0.085*	0.122*	0.023	0.038	0.023
LR χ^2^	60.64	60.93	62.51	65.48	64.46	80.94
N. obs.	2331	2331	2331	1908	1908	1908

***p < 0.001, **p < 0.01, *p < 0.05. Model 1 to model 3 are computed with samples in egalitarian regions. Models 4–6 refers to samples in non-egalitarian regions. Columns 1 and 4 refer to regular logit models; columns 2 and 5 refer to probit models; columns 4 and 6 refers to the logit model with clustering effect on residential areas (URN). There are 2,331 female observations in egalitarian regions and 1,908 female observations in non-egalitarian regions.

To sum up, our study indicated that in general, women who hold high management positions are more likely to report higher levels of happiness than those with low management positions. However, the disparities between senior and junior females are mediated by the queen bee traits and regional culture. Being a manager in less egalitarian regions is not associated with higher self-reported happiness levels. Being a manager in the egalitarian regions and supporting gender equality is associated with higher levels of self-reported happiness.

## Discussion and Conclusion

### Research Implications

Drawing on a large data set in China, this research assesses how occupational hierarchy relates to subjective happiness. Overall, our results suggest that both male and female managers report a higher level of happiness than non-managerial employees. There is well-being promoting effect for occupational hierarchy. The sub-sample analysis further suggests that context settings are of great importance. Female managers in less gender-egalitarian regions do not report a higher subjective happiness level. We also identified two types of queen bee behaviors, traditional gender role orientation and self-presenting in a masculine way. Female managers who hold traditional views on gender roles (i.e., female workers are less advantageous than the male) are associated with a lower level of happiness. Female managers who share the same preferences, values and hobbies with male counterparts report higher happiness levels.

This study makes a few significant theoretical implications. First, this study is among the first to consider both individual traits (internal factors) and social-economic environment (external factors) when investigating how QBs’ happiness is affected. The previous literature usually pays attention to either internal or external factors, and very few studies have provided a full picture of how female managers’ happiness is affected. QB is a multifaceted phenomenon, and different dimensions can influence QBs’ happiness differently. Therefore, it is significant to find out how QBs’ traits impact subjective happiness differently. For instance, female leaders who adopt a masculine approach (internal factor one) in the workplace are more likely to increase their leadership effectiveness and ultimately improve subjective happiness. On the contrary, female leaders who hold a negative attitude against gender equality (internal factor two) are less likely to gain support from their colleagues and subordinates, and, therefore, report lower happiness levels. In addition, the social-economic environment (the external factor) influences female managers’ happiness. Being a QB is not always a practical approach to avoid social identity threats. It may be associated with negative psychological payoffs (Derks et al., 2016; Scheepers and Ellemers, 2019). This study finds higher levels of happiness usually reported in egalitarian regions.

Second, it challenges the previous literature on the relationship between work-home conflict and life satisfaction by examining Chinese samples. Traditional views argue that females in high-level positions do not report higher subjective happiness due to home-work conflict in western countries (e.g., Trzcinski and Holst, 2012). Differently, this paper finds that female managers are associated with higher levels of life satisfaction in the Chinese context. As a matter of fact, the perception of work-home conflict is highly dependent on institutional settings and regional culture (Putnik et al., 2020). Chinese parents generally help alleviate the burden for women to take care of children. It leaves Chinese female managers more time to focus on work and career development (Zhao et al., 2019). Therefore, the home-work conflict may not be a serious issue for females in China. This is probably why some senior women attach more importance to job roles than family roles in China, which gives female managers higher job/life satisfaction. Besides, the traditional division of house chores is no longer prevalent in China. Both men and women actively engage in family domains and work domains.

Third, this study extends the literature (e.g., Zhao et al., 2017, 2019) by investigating the impacts of female managers’ masculine behavior on their happiness. Traditional literature analyses how female managers’ masculine behavior influences their work effectiveness while ignoring its impact on subjective happiness. To cope with the gender-discriminatory environment, females may need to display certain masculinity. For instance, they may share the same values and preferences with their male counterparts or hold more traditional attitudes. However, such masculine behavior might not necessarily generate happiness, as this behavior puts female workers at a relatively disadvantageous position compared to males. Therefore, it is interesting and valuable to understand which condition the masculinity masculine behavior will trigger happiness for female managers.

### Managerial Implications

This study has a few managerial implications. First, this study is helpful for female managers to promote their leadership effectiveness. In particular, in egalitarian regions, female managers have to highlight their masculinity to deal with social identity threats. For instance, female leaders may need to mimic their male counterparts to get approval from their subordinates. Additionally, female leaders should support gender equality than oppose it. Female managers who legitimize gender inequality usually will not gain positive psychological payoffs. Second, as previous literature suggested (e.g., Zhao et al., 2017), measures to improve gender equality should be adopted. In organizations with less egalitarian cultures, female leaders suffer from a higher social identity threat that cannot be easily avoided. Organizations need to evaluate the overall consequences of QBs before implementing any HRM practices.

### Limitations and Future Extensions

Our study is not without caveats. Due to data limitations, we cannot distinguish top managers (e.g., CEOs) from middle-level managers. Queen bees Syndrome and work-home conflict may be more evident for top executives. Second, the organizational context has been overlooked in this study. After the economic reforms, work units have been converted into profit-oriented entities which are selective in hiring employees. Females are clustered in clothes and shoe manufactures, consumer electronics, or other services industries (He and Wu, 2016). Subsequent research thus may compare the association between queen bees and happiness in both male-dominated and female-dominated industries. The significance of family composition should also be taken into considerations. Meanwhile, although we construct a gender inequality index to divide samples into different groups, one could also try other classification criteria (e.g., East and West). The disparities between East and West exist in both economic and non-economic domains, which may impact the subjective happiness of females. To make an in-depth analysis on QBs, cross-national studies are also necessary. Existing literature generally focus on single country which overlooked the cultural context. Despite some of the limitations, there are reasons why the findings of this study are noteworthy.

## Data Availability Statement

The original contributions presented in the study are included in the article/supplementary material, further inquiries can be directed to the corresponding author/s.

## Author Contributions

AX: research design and drafting. SX: drafting and revision. QW: supervision and revision. JL, HW, and HL: supervision. DC: revision. All authors contributed to the article and approved the submitted version.

## Conflict of Interest

The authors declare that the research was conducted in the absence of any commercial or financial relationships that could be construed as a potential conflict of interest.

## Publisher’s Note

All claims expressed in this article are solely those of the authors and do not necessarily represent those of their affiliated organizations, or those of the publisher, the editors and the reviewers. Any product that may be evaluated in this article, or claim that may be made by its manufacturer, is not guaranteed or endorsed by the publisher.

## Footnotes

1 We try to study the overall subjective happiness rather than the distinction between job satisfaction and life satisfaction in this study. According to the spillover hypothesis, job experiences tend to spill over onto the life domain and vice versa (Bowling et al., 2010). Therefore, numerous studies suggest a positive relationship between these two factors. For instance, based on three studies with over 2000 samples, Wenceslao et al. (2017) find that job satisfaction and life satisfaction are positively related.

2 Rankings of 2020 score can be found via https://reports.weforum.org/

3 Some researchers claim that occupational hierarchy are associated with more stress which may reduce subjective happiness (e.g., Martins and Lopes, 2012). However, they are able to gain more social and organizational support which reduces the negative impact of job stress. Therefore, we believe that the positive impact of occupational hierarchy outweighs its negative impact.

4 Explanation on methodologies of CGSS survey can be found online: http://cgss.ruc.edu.cn/English/About_CGSS/Implementation.htm

5 See the link for details about statistics: http://images3.mca.gov.cn/www2017/file/202009/1601261242921.pdf
